# The role of sulfur compounds in chronic obstructive pulmonary disease

**DOI:** 10.3389/fmolb.2022.928287

**Published:** 2022-10-19

**Authors:** Simin Jiang, Yahong Chen

**Affiliations:** Department of Pulmonary and Critical Care Medicine, Peking University Third Hospital, Beijing, China

**Keywords:** chronic obstructive pulmonary disease, hydrogen sulfide, sulfur compound, sulfane sulfur, thiols

## Abstract

Chronic obstructive pulmonary disease (COPD) is a common respiratory disease that brings about great social and economic burden, with oxidative stress and inflammation affecting the whole disease progress. Sulfur compounds such as hydrogen sulfide (H_2_S), thiols, and persulfides/polysulfides have intrinsic antioxidant and anti-inflammatory ability, which is engaged in the pathophysiological process of COPD. Hydrogen sulfide mainly exhibits its function by S-sulfidation of the cysteine residue of the targeted proteins. It also interacts with nitric oxide and acts as a potential biomarker for the COPD phenotype. Thiols’ redox buffer such as the glutathione redox couple is a major non-enzymatic redox buffer reflecting the oxidative stress in the organism. The disturbance of redox buffers was often detected in patients with COPD, and redressing the balance could delay COPD exacerbation. Sulfane sulfur refers to a divalent sulfur atom bonded with another sulfur atom. Among them, persulfides and polysulfides have an evolutionarily conserved modification with antiaging effects. Sulfur compounds and their relative signaling pathways are also associated with the development of comorbidities in COPD. Synthetic compounds which can release H_2_S and persulfides in the organism have gradually been developed. Naturally extracted sulfur compounds with pharmacological effects also aroused great interest. This study discussed the biological functions and mechanisms of sulfur compounds in regulating COPD and its comorbidities.

## Introduction

Chronic obstructive pulmonary disease (COPD) was the third leading cause of death worldwide in 2019, causing the death of 3.23 million people, which was 6% of the total death (WHO, 2019). According to a large, nationally representative cross section of adults ≥ 40 years old, the estimated standardized prevalence of COPD among the Chinese population was 13.6% (95% CI 12.0–15.2) (Fang et al., 2018). For adults ≥20 years old, the overall prevalence was 8.6% (95% CI 7.5–9.9) (Wang et al., 2018). Meanwhile, COPD was the third leading cause of disability-adjusted life-years lost in China in 2017 (Zhou et al., 2019). Moreover, driven by the aging of the Chinese population, the morbidity of COPD is expected to increase accompanied with increasing economic and social burden. Patients with COPD suffer from irreversible bronchi obstruction; however, the current therapeutic strategies, including bronchodilators, antimuscarinic drugs, methylxanthines, and corticosteroids, were adopted to alleviate the symptoms but have little effect on delaying the disease progress. Innovative drugs that can delay the disease’s progress and ideally have the potential to cure the disease are extremely needed.

Sulfur compounds include hydrogen sulfide (H_2_S), sulfur dioxide (SO_2_), organic sulfur, sulfate, and elemental sulfur, which were widely distributed in the outer environment and in organisms. For centuries, scientists believed that these compounds were responsible for damage to our environment and for causing respiratory diseases. For example, H_2_S and SO_2_ are well-known air pollutants, the high concentrations of which can cause irritation of the respiratory system, resulting in coughing, throat irritation, and shortness of breath. Sulfur mustard is an alkylating compound used as a chemical warfare agent, whose exposure led to long-term respiratory effects with several features resembling those of COPD, including chronic bronchitis, bronchial hyper-responsiveness, dyspnea with respiratory failure in advanced stages, and predominance of neutrophils in bronchoalveolar lavage fluid (Sahebkar et al., 2015).

In current years, people gave more attention to the biochemical role of sulfur compounds in the physical and pathological processes beyond the toxic effects. Sulfur is integral to the origin of life. They created an essential redox gradient that allows life to survive and evolve (Olson, 2021). The sulfur atom, with six valence electrons, can change its oxidation states from -2 to +6, thus being able to react with various nucleophiles and electrophiles to form a variety of molecular arrangements and exhibit diverse biological functions (Szabo, 2018). For COPD, oxidative stress, chronic inflammation, and protease–antiprotease imbalance are three major pathogenetic factors, of which, oxidative stress can be an initiator and amplifier for respiratory inflammation (Barnes, 2016). Reduced sulfur compounds, such as H_2_S, thiols, and sulfate sulfur (e.g., per/polysulfides), which have intrinsic antioxidant capabilities, may contribute to alleviating COPD symptoms as well as preventing COPD exacerbation (Rahman, 2012; Rahman and MacNee, 2012). Complex sulfur redox regulators, in particular, glutathione (GSH) redox buffers, sulfur-regulating enzymes superoxide dismutase, catalase, electron-conducting sulfur, and seleneosulfur enzymes, including thioredoxin, glutaredoxin, and peroxiredoxin systems, were essential in maintaining normal bioactivity in living cells (Olson, 2021). Sulfur compounds also modify bioactivity through post-transcriptional modifications, for example, S-glycosylation, S-palmitoylation, S-nitrosylation, and S-sulfidation of the cysteine residues in proteins (Ramazi and Zahiri, 2021). In addition, many pharmaceuticals consist of sulfur subunits, including mucolytic drugs, antibacterial, anti-inflammatories, antihypertensive drugs, analgesics, and anticancer agents. The effect of these sulfur compounds on the development and treatment of COPD is discussed herein.

## Hydrogen sulfide

**FIGURE 1:**
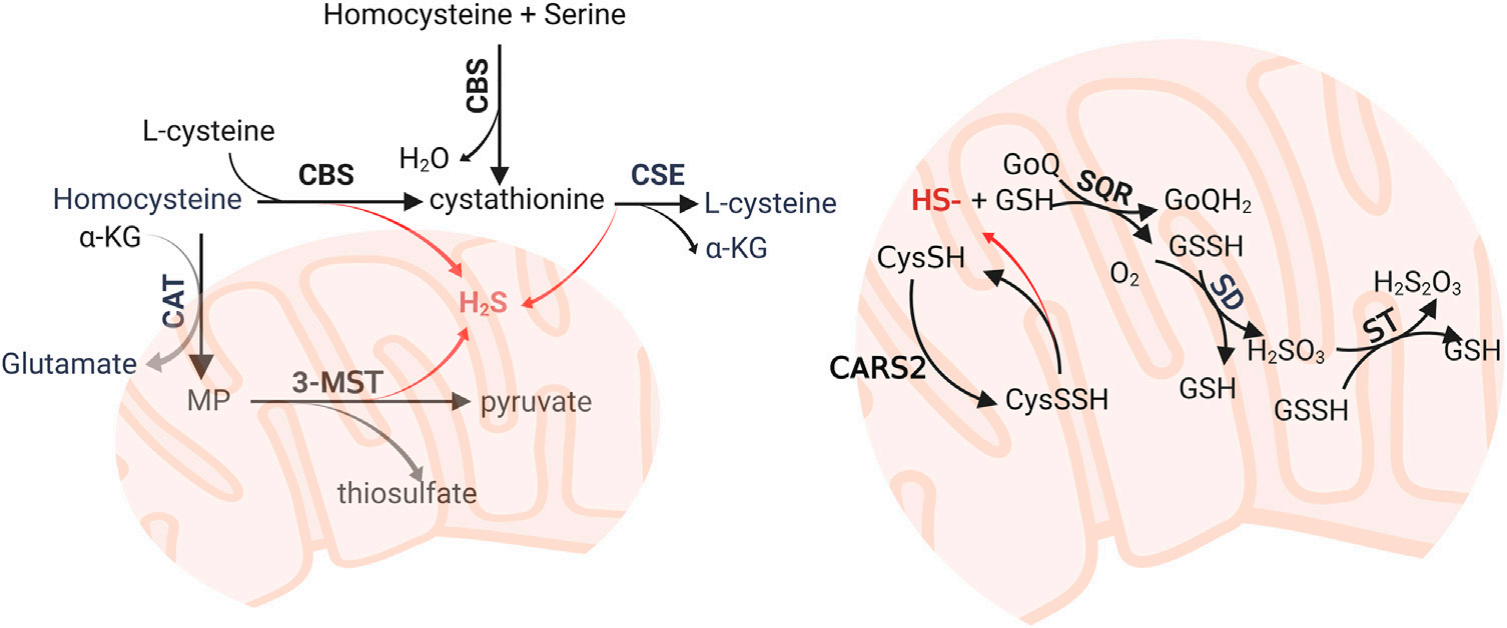
Enzymatic biosynthesis and oxidative pathway of hydrogen sulfide (H_2_S). The biosynthesis of H_2_S was mediated by CBS, CSE, and 3-MST. The major substrates are homocysteine and L-cysteine. Persulfide and polysulfides reduced in mitochondria also generate H_2_S. H_2_S can be oxidized by SQR, SD, and ST, which are mitochondrial inner membrane-anchored enzymes, to form sulfate and deprived from the organism. CBS: cystathionine β-synthase; CSE, cystathionine γ-lyase; CAT, cysteine aminotransferase; CoQ, coenzyme Q; CARS, cysteinyl-tRNA synthetases; 3-MST, 3-mercaptopyruvate; MST, 3-mercaptopyruvate sulfurtransferase; α-KG, α-ketoglutarate; SQR, sulfide quinone reductase; SD, sulfur dioxygenase; ST, sulfur transferase.

H_2_S is the an endogenous gas transmitter, along with nitric oxide and carbon monoxide. Endogenous H_2_S can be synthesized *via* enzymatic and non-enzymatic pathways. The enzymatic pathway is mediated by cystathionine-γ-lyase (CSE), cystathionine-β-synthase (CBS), and mercaptopyruvate sulfurtransferase (MST) (Li et al., 2011) (Figure 1). The non-enzymatic pathway includes the reduction of elemental sulfur to H_2_S using reducing equivalents obtained from the oxidation of glucose (Wang, 2002). Intracellular sulfane sulfur also formed an H_2_S storage pool. CSE and CBS are located in the cytosol. When faced with stimuli, they can translocate to mitochondria to enhance H_2_S production (Fu et al., 2012; Teng et al., 2013). MST is combined with cysteine aminotransferase (CAT) to synthesis H_2_S in mitochondria. In the human lung tissue, they are expressed in the alveolar cells and endothelial cells based on the Protein Atlas Database (Olson et al., 2010; Pacitti et al., 2021). The deprivation of H_2_S is mediated by sulfide quinone reductase (SQR) in the mitochondria, where H_2_S was oxidized into sulfate and finally removed from the kidney (Libiad et al., 2019; Landry et al., 2021). H_2_S can also be directly exhaled *via* the airway.

Impaired lung growth during gestation and childhood would reduce maximal attained lung function, which puts individuals at risk of developing COPD (Stern et al., 2007). Disturbing of CBS and CSE expression led to muscularization of small- and medium-sized lung vessels and incomplete lung alveolarization during fetal lung development, while exogenous introduced H_2_S improved alveolarization (Madurga et al., 2015). CSE was downregulated in smokers and COPD patients, but CBS mRNA transcript was increased in smokers and decreased in COPD patients when compared with healthy controls (Sun et al., 2015). H_2_S can be a valuable biomarker indicating the development of COPD and reflecting the disease states. Endogenous H_2_S can be detected in blood, sputum, and exhaled gas. Serum H_2_S was increased in patients with stable COPD when compared to healthy controls and acute exacerbation of COPD (AECOPD), and its concentration was positively correlated with the lung function of COPD patients (Chen et al., 2005). Low serum H_2_S was associated with respiratory tract infection, whose receiver operating characteristic curve for predicting the need for antibiotic treatment for COPD patients was 0.862 (Chen et al., 2009). H_2_S is involved in vascular remodeling in COPD, and its concentration was negatively correlated with indexes like main pulmonary artery diameter on HRCT, those indirectly reflecting the pulmonary artery tension (Liao et al., 2021). However, serum H_2_S was affected by systemic metabolism; for example, H_2_S is synthesized by bacteria in the gut or released from other organs, thus being less specific to identifying lung diseases. H_2_S in sputum was considered to more closely reflect lung diseases. An increase of the sputum-to-serum ratio of H_2_S was found among AECOPD subjects (Saito et al., 2014). Moreover, the ratio is positively associated with sputum neutrophils, both in COPD and in asthma (Saito et al., 2014; Suzuki et al., 2018). Exhaled H_2_S is a valuable clue to respiratory diseases since it changes dynamically with disease states and can be measured noninvasively. Current studies did not find the exhaled H_2_S differing significantly from stable COPD, AECOPD, and healthy groups. However, its concentration was negatively correlated with induced sputum eosinophils, thus being regarded as a biomarker that indicates a non-eosinophilic disease phenotype (Sun et al., 2013; Zhang et al., 2015). However, the result was not very conclusive due to the limited sample size. In addition, this study collected the exhaled H_2_S using reservoir bags to carry out off-line detection, which was not very competent in identifying the real-time changes. As a result, a large-scale, on-line survey of exhaled H_2_S among COPD patients and healthy controls is still needed to eliminate numerous confounding factors affecting H_2_S exhalation.

**FIGURE 2:**
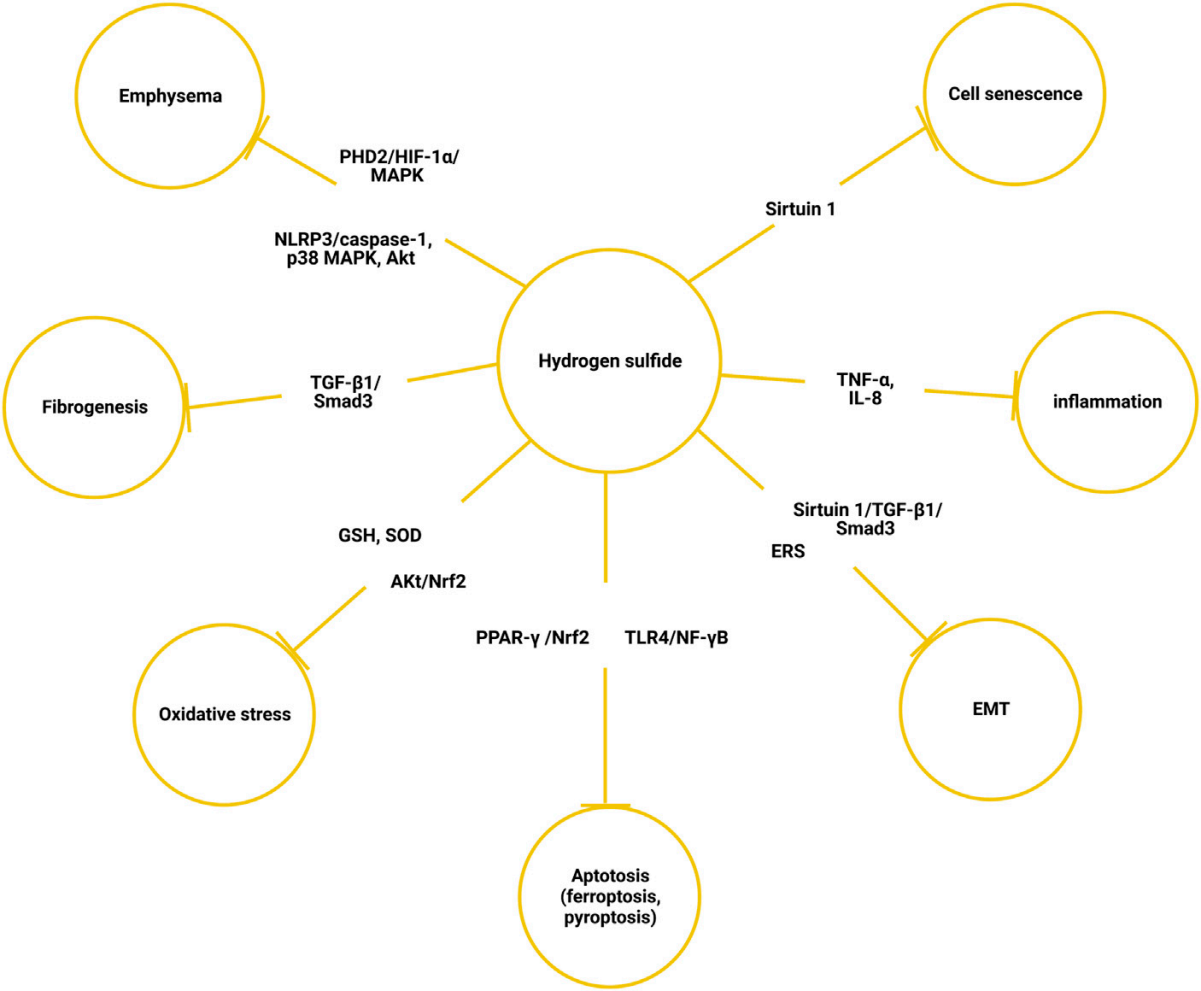
Mechanism of hydrogen sulfide alleviating chronic obstructive pulmonary disease. EMT, epithelial–mesenchymal transition; ERS, endoplasmic reticulum stress; SOD, superoxide dismutase.

Hydrogen sulfide is a novel gasotransmitter involved in a variety of bioactivities, such as vasodilation, antioxidant, anti-inflammation, mesenchymal transition, cell senescence, and apoptosis. H_2_S is a vasodilator, and its consumption may be O_2_-dependent as a mediator of the hypoxic pulmonary vessel constriction (Olson et al., 2010). Apart from being a vasodilator, the anti-inflammation and antioxidant effects of endogenous H_2_S have been well-documented, both in COPD and other diseases (Bhatia, 2012). (Figure 2) Exogenously introduced NaHS, a commonly adopted H_2_S donor, can protect against cigarette smoke-induced oxidative stress by promoting Akt phosphorylation. Consequently, the antioxidant transcription factor Nrf2 was upregulated, which otherwise would be inhibited by cigarette smoke. The pathological manifestation was also improved in the mouse model, with ameliorated bronchial remodeling, lung emphysema, and pulmonary hypertension (Han et al., 2011). The protective effect of H_2_S against particulate matter-induced emphysema and airway inflammation is exhibited *via* the Nrf2-dependent pathway through suppressing PYD domain-containing protein 3 (NLRP3) inflammasome formation and apoptosis (Jia et al., 2020). The H_2_S donor, GYY4137, promoted the production of GSH and superoxide dismutase and inhibited the release of inflammatory factors like TNF-α and IL-8 (Sun et al., 2015). H_2_S inhibited the transform growth factor beta-1 (TGF-β1)/Smads signaling pathway, which was associated with pulmonary fibrogenesis and airway remodeling of COPD (Jerzy et al., 2009; Liao et al., 2015; Wang et al., 2020). Airway epithelial–mesenchymal transition (EMT) is a highly plastic process through which epithelial cells change into a mesenchymal phenotype following epithelial damage. In human bronchial epithelial cells, NaHS treatment upregulated sirtuin 1 expression, which modified TGF-β1-mediated Smad3 transactivation, and then cigarette smoke extract-induced EMT, collagen deposition, and oxidative stress was reduced (Bhatia, 2015; Guan et al., 2020a). As a nicotinamide adenine dinucleotide (NAD^+^)-dependent histone deacetylase, sirtuin 1 plays a central role in cell senescence and aging (Xu et al., 2020). H_2_S upregulated sirtuin 1 expression, which improved mitochondrial function and reduced oxidative stress; thus, cell senescence induced by cigarette smoke was attenuated (Guan et al., 2019). Hydrogen sulfide also inhibits EMT by regulating endoplasm reticulum stress (ERS) (Lin et al., 2022). With ERS markers such as glucose-regulated protein-78 (GRP78), C/EBP homologous protein (CHOP), and caspase-12 were reduced in the COPD rat model that was established by passive smoke exposure and lipopolysaccharide irritation, pulmonary artery endothelial cell apoptosis was decreased (Ding et al., 2018). In a cigarette smoke-exposed rat model, H_2_S also inhibited ERS and apoptosis in bronchial epithelial cells (Lin et al., 2017). NaHS prevented emphysema *via* the suppression of the PHD2/HIF-1α/MAPK signaling pathway, and subsequently inhibition of inflammation, epithelial cell injury, and apoptosis (Guan et al., 2020b). Apart from cigarette smoke-induced COPD, H_2_S also alleviates lung emphysema induced by other etiologies. For example, NaHS administration prevented and partially reversed ozone-induced features of lung inflammation and emphysema *via* the regulation of the NLRP3-caspase-1, p38 MAPK, and Akt pathways (Li et al., 2016). A recent study found that H_2_S attenuates cigarette smoke-induced pyroptosis through inhibition of TLR4/NF-κB signaling (Wang L. et al., 2022). Ferroptosis was found to be elevated in COPD. Administration of NaHS can alleviate particulate matter-induced emphysema and airway inflammation by suppressing ferroptosis *via* PPAR-γ/Nrf2 signaling (Wang Y et al., 2022).

Researchers usually introduced H_2_S to an animal model simultaneously or before harmful factors like cigarette smoking, lipopolysaccharide, and ozone exposure was introduced so that the preventive effect of H_2_S on the development of COPD was fully confirmed. However, its therapeutic potential to reverse bronchial remodeling or airway obstruction was still uncertain. One research showed that H_2_S inhibited phosphorylation of the MAPKs, extracellular signal-regulated kinase (ERK)-1/2, and p38 to regulate airway smooth muscle cell proliferation and cytokine release (IL-6 and CXCL8) among nonsmokers. However, in cell lines obtained from COPD patients, these protective processes were disturbed (Perry et al., 2018). As a result, whether exogenous administration of H_2_S can delay the disease progress after COPD was exhibited was under doubt. Moreover, most of the studies used exogenous H_2_S donors as an intervention in the biosystem, but the concentration of H_2_S generated from H_2_S donors is much higher than its physiological concentration (Pacitti et al., 2021). The huge gap between the level of exogenous introduced H_2_S and the endogenous circulated H_2_S raised a concern whether endogenous H_2_S has similar bioactivity with the exogenous introduced H_2_S.

## Thiol-based family

Thiols are organosulfur compounds with the general formula R-SH, where R represents an alkyl or other organic substituent. They can be oxidized by reactive oxygen species (ROS) to form disulfide or sulfate. Thiols’ redox states reflect the oxidative stress in the organism, of which the GSH redox couple is the major non-enzymatic redox buffer that accounts for approximately 90% of intracellular small molecular thiols (Heffner and Repine, 1989; Sotgia et al., 2020). The disturbance of the redox state was observed in a variety of respiratory diseases, such as cystic fibrosis, acute respiratory distress syndrome, asthma, and COPD (Rahman and MacNee, 1999; Zinellu et al., 2016). In patients with COPD, increased oxidative stress and reduced GSH have been found in biofluids like exhaled breath condensate, sputum, and blood (van der Toorn et al., 2007). Epithelial lining fluid is an aqueous continuous layer with high GSH concentration. It covers the mucus of the airway and alveoli to defend against exogenous oxidants that are contained in tobacco and air pollution. The total GSH in the epithelial lining fluid is up to 140-fold higher than that in plasma such that 90% of them are in reduced form (Cantin et al., 1987). Smoke induced a rapid decline of GSH, but chronic exposure led to increasing GSH in the lung epithelial lining fluid to mount a protective response (Gould et al., 2011). Aging eliminated the adaptive response from GSH when compared to younger controls (Gould et al., 2010). In addition, the lowered plasma cysteine/cystine ratio of COPD is correlated with the extent of loss of lung function (Watson et al., 2019). It was reported that cigarette smoking can deplete the total GSH pool by oxidizing GSH to nonreducible GSH-aldehyde derivatives (van der Toorn et al., 2007). According to a meta-analysis that involved 18 studies with 974 COPD patients and 631 healthy groups, the pooled reduced GSH in blood was significantly lower in COPD than in controls, although the total GSH concentrations were increased (Sotgia et al., 2020). However, another meta-analysis including 14 articles of 902 COPD patients and 660 controls reported that the total GSH was not significantly different between patients and controls, and pooled reduced GSH concentrations showed either a significant or nonsignificant difference depending on whether the mean concentrations of reduced GSH in controls were correctly within the accepted normal range (0.5–5.0 umol/L) (Sotgia et al., 2021). Methodological factors vastly affect the measurement of GSH. For instance, reduced GSH can undergo autoxidation *in vitro* and present an artificial oxidative state. Also, some studies falsely measured reduced GSH by spectrophotometric methods using Ellman’s reagent because this reagent reacted with sulfhydryl compounds rather than specifically to reduced GSH (39). In fact, many small-molecular thiols like cysteine and homocysteine can react with oxidants and are functionally close to GSH; therefore, a comprehensive redox state analysis of thiols may be better in the evaluation of systemic oxidative stress in COPD.

N-acetylcysteine, carbocysteine, and erdosteine are thiol-based mucolytic drugs. N-acetylcysteine contains a free SH group. Carbocysteine and erdosteine are thiol derivatives that can produce sulfhydryl compounds *via* metabolization. They disturbed the disulfide bonds in proteins to decrease the viscosity and elasticity of the mucus (Cazzola et al., 2020). N-acetylcysteine can replenish reduced GSH shortage by supplementing cysteine *via* deacetylate in the gastrointestinal tract, and cysteine is the rate-limiting substrate for GSH synthesis (Atkuri et al., 2007; Rahman and MacNee, 2012). Apart from mucolytic activity, multiple pharmacological activities of these drugs have been documented (Cazzola et al., 2019; Cazzola et al., 2020). Thiol-based drugs can directly scavenge reactive oxidative species and reactive nitrogen species *via* reducing equivalent free SH group and indirectly suppress the oxidative stress *via* modulating neurokinin A levels. The anti-inflammatory effects were exhibited by their reducing the synthesis and release of cytokines, proteinases, and proinflammatory factors, as well as inhibiting neurogenic inflammation. They can also reduce bacterial adhesion to the airway epithelial cells and disturb biofilm formation, thus being auxiliary drugs to improve antibiotic activity. Moreover, thiol-based drugs can even regulate the tone of airway smooth muscles in human bronchi. Carbocysteine can restore steroid sensitivity by increasing histone deacetylase 2 expression in a thiol/GGSH-dependent manner (Song et al., 2015; Song et al., 2019). Thiols regulated autophagy augmentation, whose impairment led to emphysema of COPD (Vij et al., 2018; Bodas et al., 2019).

Traditionally, mucolytic drugs were used during COPD exacerbation at low dosage and for a short period of time. Recently, some evidence indicated that using these drugs in high dosage and in the long term can prevent COPD progression and exacerbation. Bridgeman et al. (1994) reported a dose-dependent effect of N-acetylcysteine administration on plasma GSH in COPD. N-acetylcysteine did not increase the plasma GSH at 600 mg daily until 600 mg, three times a day. Regular usage of N-acetylcysteine (1200 mg daily) can reduce COPD exacerbations, especially for patients with heavy smoking history and for those who did not receive ICS treatments (Papi et al., 2019). A meta-analysis included seven randomized clinical trials with 2,753 patients confirmed the safety and efficacy of regular using mucolytic drugs in COPD (Rogliani et al., 2019). Erdosteine (600 mg/day), carbocysteine (1500 mg/day), and N-acetylcysteine (1200 mg/day) can reduce the risk of exacerbation and hospitalization with few adverse events. The ranks of effectiveness were erdosteine > carbocysteine > N-acetylcysteine. Erdosteine even prevented mild exacerbation, irrespective of concurrent ICS treatment. However, the latest Global Initiative for Chronic Obstructive Lung Disease strategy’s approach (GOLD, 2022) did not suggest antioxidant mucolytic drugs being used constitutively in the management of stable COPD (GOLD, 2022). More evidences were needed to identify the selected subjects who would benefit from this strategy. The benefits and defects of long-term treatment of mucolytic drugs among patients with COPD should be further explored.

## Sulfane sulfur

**FIGURE 3:**
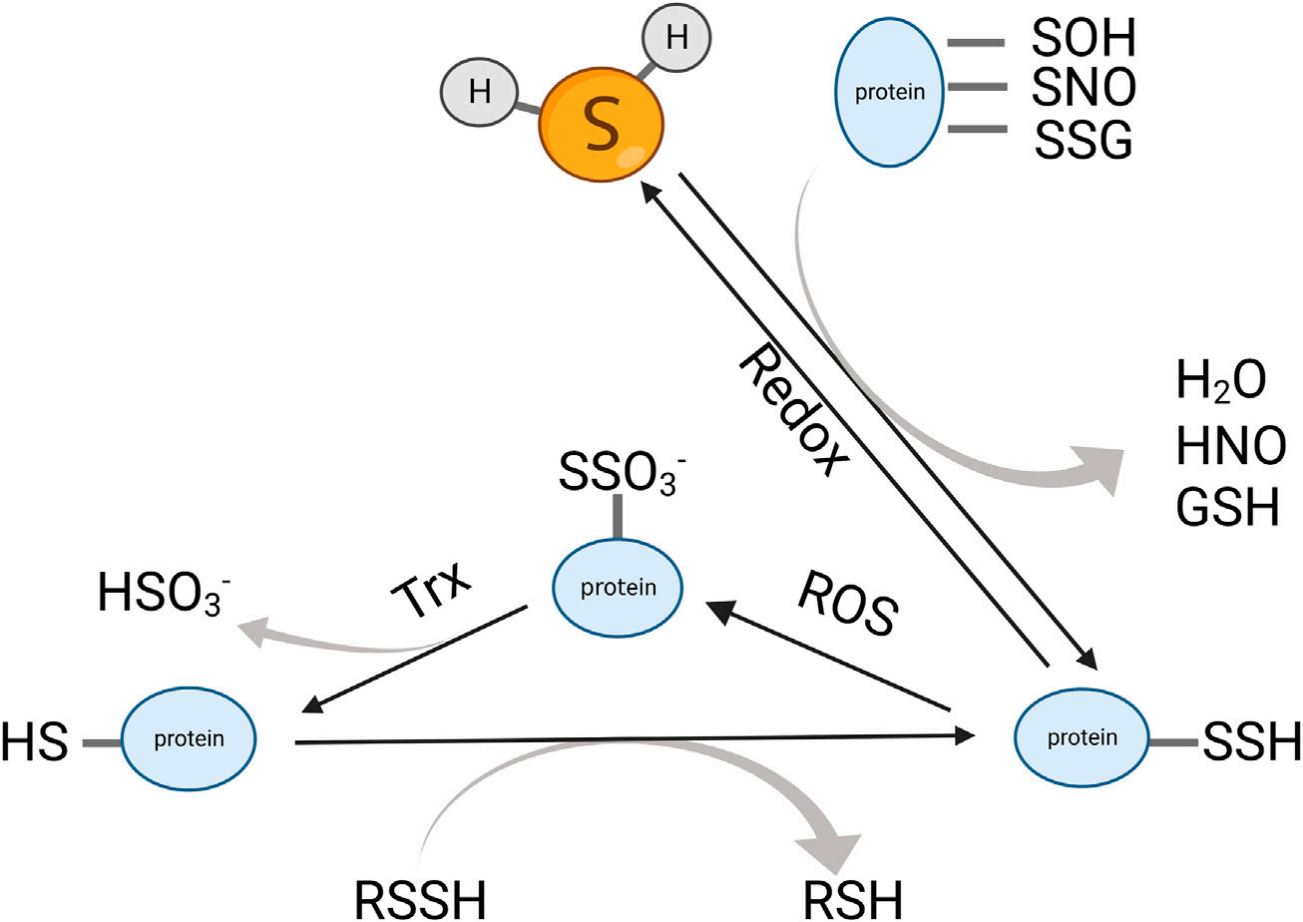
Dynamic interchange between protein persulfides, protein thiols, and H_2_S. H_2_S can react with an oxidized cysteine residue in protein (e.g., -SOH, -SNO, and -SSG) to generate protein persulfides. The protein persulfides can be oxidized by ROS and subsequently reduced by Trx to form thiols. The protein thiols can also transform into protein persulfides *via* trans-sulfidation. Trx, thioredoxin; ROS, reactive oxygen species.

Sulfane sulfur refers to a divalent sulfur atom bonded with another sulfur, such as inorganic (H_2_Sn) or organic persulfides (RS_n_H or RS_n_R, *n* = 2) and polysulfides (*n* = 3–8). It also includes disulfides where the C–S bond is adjacent to an unsaturated bond, for example, C=C or C=O, because the unsaturated bond near the C-S can tautomerize to a thiosulfoxide that contains a sulfur atom with six electrons (Iciek et al., 2001). Unlike thiols that can only act as reductants, sulfane sulfur is both nucleophile and electrophile, which can react with a variety of compounds to exert its biological activities. The numerous intracellular sulfane sulfur compounds formed an H_2_S storage pool to maintain a reduced environment in the cytoplasm. Persulfide/polysulfide derives from H_2_S oxidation or reacts with nitric oxide (NO) (Figure 3) (Ida et al., 2014). CBS and CSE catalyze CysSSH biosynthesis using cystine (CysSSCys) as a substrate, and they play a role in the trans-sulfuration pathway along with rhodanese (Koj and Frendo, 1962; Iciek et al., 2019). However, some evidences showed that CSE and CBS do not contribute directly to persulfide production but may promote the biosynthesis of cysteine and its supply to cysteinyl-tRNA synthetases (CARS) (Akaike et al., 2017). CARS can incorporate CysSSH into the protein during translation. In addition to their canonical role in protein translation, CARS also act as the principal cysteine persulfide synthases *in vivo*, which catalyzed both low-molecular-weight polysulfides and polysulfidated proteins. Notably, CARS2, a mitochondrial isoform of CARS, is involved in mitochondrial biogenesis and bioenergetics *via* CysSSH production, and the cysteine persulfide and polysulfides that are generated by CARS2 were important resources for production of H_2_S.

Protein persulfidation is a recently recognized posttranslational modification. Sulfhydration can protect the proteins from oxidation and keep them in a more stable state. It usually enhances protein activity. Protein persulfidation was a major way that H_2_S exhibited its biological function. In 2009, Asif K. Mustafa reported that H_2_S can directly sulfhydrate the cysteine residue in GAPDH (Mustafa et al., 2009). After that, other target proteins of H_2_S with the cysteine residue have gradually been reported, such as Keap1, sirtuin 1, and ATP5A (Yang et al., 2013; Modis et al., 2016; Du et al., 2019). However, direct sulfhydration cannot be observed in a cell-free system. Some suppose that H_2_S cannot directly react with proteins that contain cysteine residues. This posttranslational modification is initiated by H_2_S reacting to oxidized protein thiols or protein thiols reacting to reactive sulfur species generated from oxidized H_2_S (Stubbert et al., 2014; Beltowski, 2015). Other studies found that H_2_S can break down disulfide bonds in proteins to exert biological function. For example, VEGFR2, insulin receptor, and EGFR underwent a disulfide bond molecular switch when reacted with H_2_S (Tao et al., 2013; Xue et al., 2013; Ge et al., 2014).

Protein persulfidation has been widely studied in the cardiovascular system, gastrointestinal system, neuron system, and immune system. However, few studies revealed the role of persulfidation relative to COPD. Persulfidation was confirmed to be an evolutionarily conserved modification with antiaging effects, and aging is a major risk factor for COPD. Zivanovic et al. (2019) revealed that the overall persulfidation increased in the early developmental stage, then hit a plateau, and finally declined in the late stage of growth, and this process was irrespective of species specificity. Increased persulfidation was associated with increased longevity and improved capacity to cope with stress stimuli. Furthermore, the level of persulfides and polysulfides as GSSH, CySSH, and GSSSH decreased in the epithelial lining fluid and primary lung cells of the patients with COPD when compared to healthy controls (Numakura et al., 2017). The reactive persulfides and polysulfide decreased significantly among patients with asthma-COPD overlap disease, when compared to the asthma group and healthy controls (Kyogoku et al., 2019). However, the persulfidation signaling pathway that is specifically related to COPD pathophysiology was not fully recognized, and more research is needed to identify the specific persulfidation signaling pathway involved in COPD.

## Assessment of sulfur compounds

Accurate identification and assessment of sulfur compounds are the foundation for analyzing their biological functions. However, it is not simple as the sulfur compounds are under dynamic changes, so the measurement reflects a cross-section of the transient changes. Some of them have similar biochemical reactivities, which cause false-positive or false-negative results. Moreover, H_2_S would be auto-oxidized when it was exposed to oxidants in the air so that the precision of the result is affected by decay between the time of blood collection and sulfide measurement. There were many strategies for H_2_S detection, including spectroscopic, chromatographic, and electrochemical methods (Ibrahim et al., 2021). Each has advantages and limitations regarding the sample to be measured. For example, the methylene blue method was associated with strong acidic conditions, while H_2_S will be released from sulfane sulfur and acid-labile sulfur pool under the influence of reductants or strong acids, therefore resulting in overestimated H_2_S concentration (Li and Lancaster, 2013). The gas-sensing electrode is an electrochemical method that can detect sulfur ions with a sensitivity of 1–100 lM, but it is only sensitive to dissociated S^2-^, which is present under alkali and oxidation-free conditions (Cao et al., 2019). However, the alkaline conditions of the antioxidant buffer induce the lability of protein sulfur and produce artificially elevated sulfide values (Olson, 2009). An alternative method for electrochemical measurement of sulfide was the polarographic H_2_S sensor (Doeller et al., 2005; Whitfield et al., 2008). It permits real-time measurement of H_2_S gas in biological fluids without sample modification, while dissolved H_2_S (HS^−^ and S^2-^) is estimated indirectly within the knowledge of pH and PKa (Whitfield et al., 2008). Fluorogenic probes can be adopted to visualize relative changes in H_2_S concentration *in vivo*, with a high spatiotemporal resolution of signals at the cellular and organelle level (Yu et al., 2013; Ibrahim et al., 2021). However, the results were diverse across imaging techniques and tissue autofluorescence and affected by different rates of uptake and retention of fluoroscopy dye across cell types, which makes it incomparable between different studies (Pacitti et al., 2021). Chromatography is a useful method for H_2_S detection with high sensitivity and specificity. It includes gas chromatography coupled to detectors as electrochemical, electron capture, flame photometry, mass spectrometry, ion mobility spectrometry, and liquid chromatography coupled to detectors as spectrophotometry, spectrofluorimetric, atomic fluorescence spectrometry, mass spectrometry, and electrochemical (Ibrahim et al., 2021). Chromatography can distinguish sulfide in different biochemical forms, including acid-labile sulfur and free and bound sulfane pools. With different strategies being applied, the physiological level of H_2_S in the tissue has not reached a consensus, with specifically some reported at the level of μM, and some confirmed to be at the level of nM (Furne et al., 2008; Whitfield et al., 2008).

The methods for detection of S-sulfhydration were continued to be modified. The Modified Biotin Switch Assay was derived from a nitrosylation assessment. The technology uses thiol-blocking agents, e.g., S-methyl methanethiosulfonate (MMTS) and S-4-bromobenzyl methanethiosulfonate (BBMTS), to block free thiols in the first step and then label the unreacted persulfide with N-[6-(biotinamido) hexyl]-3’-(2′-pyridyldithio) propionamide (biotin-HPDP). Finally, biotinylated protein was immunoprecipitated by Western blotting (Mustafa et al., 2009; Pan and Carroll, 2013). However, thiols have similar reactivity to MMTS with the persulfide group, which causes overestimation of persulfides. Another route is to block both thiols and persulfides with electrophiles (e.g., iodoacetic acid, IAP) and then use DDT to reduce persulfides and relabel them with biotinylated IAP (Krishnan et al., 2011). However, the specificity is under doubt because other oxidized cysteines such as disulfide bonds, sulfenic acids, and nitrosothiols can also be reduced by DDT (Krishnan et al., 2011; Pan and Carroll, 2013). The biotin thiol assay (BTA) labeled the reactive -SH and -SSH with a biotin-conjugated maleimide, and then they were bound on an avidin column. Finally, DTT was adopted to elute the retained proteins that contain a persulfide bridge (Gao et al., 2015). Protein persulfide being pulled down can be further analyzed by Western blot. The ProPerDP method has a similar mechanism as the BTA method (Doka et al., 2016). The tag switch assay first uses methylsulfonyl benzothiazole (MSBT) to block both -SH and -SSH, while the adducts resulting from persulfides are disulfides that can react with carbon-based nucleophiles (Park et al., 2015). Therefore, a cyanoacetate-based reagent CN-biotin can be introduced to be a “switch tag.” Recently, a dimedone-based probe switching method has been reported with high chemo-selectivity (Zivanovic et al., 2019). First, 4-chloro-7-nitrobenzofurazan (NBF-Cl) was applied to label persulfides, thiols, sulfenic acids, and amino groups and transform persulfides into mixed aromatic disulfides. Next, the NBF tag on persulfides is selectively switched by a dimedone-based probe. This method can enable both proteomic analysis and intracellular visualization of persulfides. Many fluorescent probes selectively reporting -SSH have been developed for detecting persulfides, poysulfides, and elemental sulfur (Chen et al., 2013; Takano et al., 2016; Bibli et al., 2018; Meng et al., 2018; Neill et al., 2019; Ran et al., 2019). Resonance synchronous spectroscopy (RS_2_) has been used to detect reactive sulfane sulfur, which displayed species-specific RS_2_ spectra (Li et al., 2019). The protonated form of persulfide (RSSH) was electrophilic and produced an RS_2_ signal, while RSS- was nucleophilic with no RS_2_ signal. Both the fluorescent probes and the resonance synchronous spectroscopy method enable quantitative measurement of reactive sulfane sulfur inside the cell or in the cellular subcompartment with high sensitivity and specificity. However, the major defect is that they do not allow qualitative detection of the sulfane sulfurs.

## Cross-talk with nitric oxide

**FIGURE 4:**
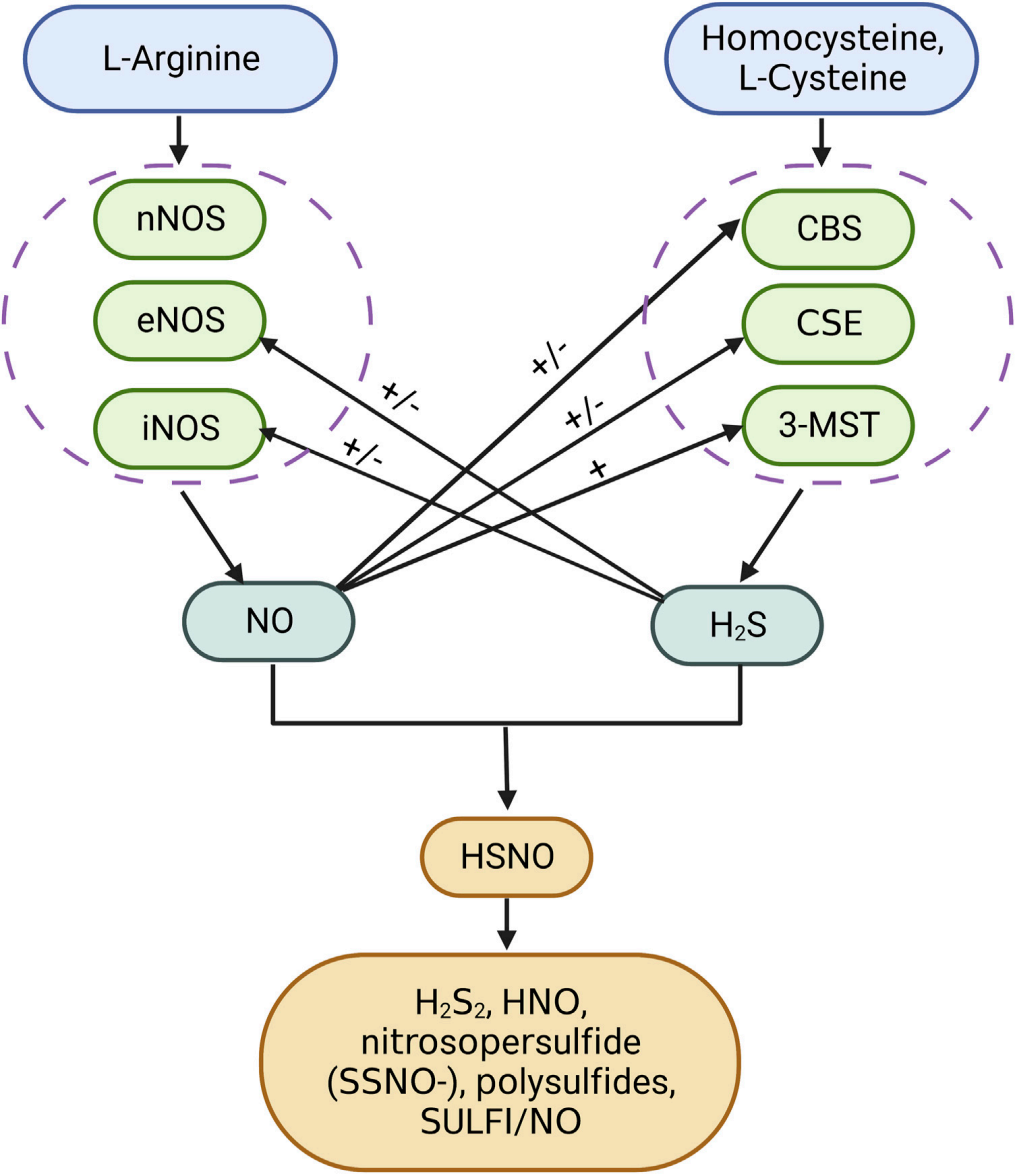
Interaction between hydrogen sulfide and nitric oxide. H_2_S can directly react with NO to form HSNO. HSNO is unstable, which can be reduced by H_2_S to form HNO and H_2_S_2_. Chemical interplay of the H_2_S donor Na_2_S with the NO donor (DEA/NO or RSNOs) produces nitrosopersulfide (SSNO-), polysulfides, and SULFI/NO. H_2_S has both activation and inhibition ability toward the synthesis of NO and *vice versa*. SULFI, N-nitrosohydroxylamine-N-sulfonate.

Airway inflammation is an important characterization for the COPD phenotype. Patients with COPD and airway eosinophilia were more sensitive to corticosteroid therapy than patients with non-eosinophilia inflammation. Elevated exhaled NO and high blood eosinophil count (≥300 cells/µL) are useful biomarkers that predict airway eosinophilia (Annangi et al., 2022; GOLD, 2022). NO is generated from guanidine nitrogen of L-arginine under the catalysis of the NO synthase (NOS) family, including endothelial (eNOS), inducible (iNOS), and neuronal (nNOS) (Gantner et al., 2020). The nNOS existed in the neurons and eNOS was most abundant in endothelial cells. iNOS was induced by inflammatory factors and expressed in inflammatory cells, while in the lung epithelium, there is a constitutive expression of iNOS (Bayarri et al., 2021). The biosynthesis of H_2_S was intertwined with NO production (Figure 4). A previous study indicated that exhaled H_2_S was positively correlated with exhaled NO in patients with COPD and healthy controls (Sun et al., 2013). H_2_S enhances the activity and expression of eNOS (Li et al., 2017). CSE deficiency in mice causes eNOS dysfunction, NO reduction, and aggravated myocardial ischemia/reperfusion injury (137). On the other hand, H_2_S restricted NO activity by forming a nitrosothiol compound, which inhibits eNOS in smooth muscle cells (Skovgaard et al., 2011). The regulation of H_2_S generation by NO is very complicated. NO was reported to enhance endogenous H_2_S production by promoting CSE expression and facilitating the uptake of L-cysteine as a substrate for H_2_S production in vascular endothelial cells (Li et al., 1999; Wang, 2012). In a pulmonary hypertensive rat model, L-arginine treatment elevated plasma H_2_S concentration, H_2_S production rate, and CSE mRNA expression in lung tissues. Meanwhile, there were contradictory findings stating that NO had no influence on H_2_S synthases nor inhibits CBS activation (Chen et al., 2014; Vicente et al., 2016). In lung cancer cells, NO can suppress CBS activity by oxidizing its ferric heme subunit (Wang and Yang, 2016). The crosstalk between H_2_S and NO was cell-specific and concentration-relevant. More research studies are needed to explore the relationship between H_2_S and NO in lung tissue during physiological and pathological states.

## Sulfur molecules and comorbidities of COPD

The disturbance of the redox buffer makes the patients more vulnerable to oxidative stress, and this may be the reason for consistent systemic inflammation presented in patients with COPD. Systemic inflammation plays an important role in the development of multiple comorbidities. Cardiovascular diseases are very prevalent comorbidities in COPD. Elevated plasma homocysteine concentration has long been recognized as an independent risk factor for cardiovascular diseases (Ozkan et al., 2002). The trans-sulfuration pathway is the major way for homocysteine clearance, and during this process, a range of low-molecular thiols are produced, which act as the major antioxidant buffers *in vivo*. The concentrations of plasma homocysteine and cysteinylglycine were elevated in patients with COPD (Zinellu et al., 2020), which was significantly associated with abnormal lung function parameters as well as COPD severity, and the disturbance of small-molecule thiols may be involved in the increased cardiovascular risks among COPD patients. Patients with COPD and cardiovascular diseases have lower H_2_S and homocysteine levels than those in the COPD group (He et al., 2017). In addition, endogenous H_2_S plays a cardioprotective role in regulating heart rhythm, modifying cardiovascular remodeling, hypertension, and atherosclerosis (Ma et al., 2015; Meng et al., 2015; Xie et al., 2016; Watts et al., 2021). Disturbance of H_2_S and other sulfur-containing molecules could be initiator factors for the development of cardiovascular comorbidities among patients with COPD.

Obstructive sleep apnea (OSA) is a disorder characterized by repeated hypopnea and hypoxemia during sleep. Patients with OSA-COPD overlap syndrome experience more frequent hypoxemia and cardiac arrhythmias (Shepard et al., 1985; Chaouat et al., 1995). The oxygen homeostasis is sustained by a hypoxic ventilatory response, which is regulated by the carotid body, a peripheral arterial chemoreceptor for O_2_ sensor. Smoke can inhibit hypoxic ventilatory response and induce aggravated hypoxemia during sleep, especially in relevant clinical conditions such as COPD (Hildebrandt et al., 2016). The thiol/disulfide redox state in the plasma and in peripheral blood mononuclear cells could massively affect the hypoxic ventilatory response *via* affecting carotid body O_2_ chemosensitivity (Lipton et al., 2001; Hildebrandt et al., 2002). It is reasonable to infer that the thiol/disulphide redox state in COPD is one of the reasons for lower hypoxic ventilatory response, which leads to aggravated hypoxemia during sleep, especially in patients with OSA-COPD overlap syndrome.

Lung adenocarcinoma expressed high levels of CBS, CSE, and MST relative to adjacent lung tissue (Szczesny et al., 2016). H_2_S synthesized by these enzymes can promote mitochondrial DNA repair and bioenergetics. The enhanced H_2_S synthesis also protects the cancer cells from therapeutic drugs. Single-nucleotide polymorphisms in the CBS [CBS rs2850146 (-8283G > C)] were significantly associated with high methylation in males (Flores et al., 2012). The variant allele of rs2850146 was associated with increased plasma homocysteine concentrations and gene hypermethylation, a known biomarker for promoting lung cancer (Flores et al., 2012), while organosulfur compounds, for example, isothiocyanates, allyl compounds, and sulforaphane, can inhibit the reactivity of histone deacetylase inhibitors and induce histone hyperacetylation in cancer cells, resulting in elevated p21 protein expression and cell cycle arrest (Nian et al., 2009).

## Innovative drugs derived from sulfur compounds

Although numerous research studies demonstrated the therapeutic potential of H_2_S, the pharmacological application of H_2_S was limited by its gaseous nature and toxicity at high concentrations. Selective and controllable release of H_2_S is essential for its therapeutic application. To solve this problem, people constructed H_2_S donors that were triggered by hydrolysis, thiols, which provide a more controllable release (Powell et al., 2018a; Zheng et al., 2018). Synthetic compounds that release H_2_S upon activation by external stimuli such as light, reactive oxygen species, and enzymes were also been developed (Zheng et al., 2016; Chauhan et al., 2017; Xiao et al., 2017). External stimuli can induce spatial and/or temporal control of H_2_S release. For example, using redox-activated metals, such as Cu, Pt, Co, Fe, Ru, Os, and Ir, can develop prodrugs that are specifically activated in hypoxic cells, and then the H_2_S can be selectively delivered into cancer cells or ischemic cells (Woods and Wilson, 2021). Some prefer the delivery of persulfides or polysulfides to direct protein S-sulfidation. Persulfides that are triggered by PH, esterase, reactive oxygen species, and photons were gradually been developed, for instance, a hydrogen peroxide-sensing motif was constructed to develop a reactive oxygen species-activated persulfide donor (Artaud and Galardon, 2014; Powell et al., 2018b; Yu et al., 2018; Yuan et al., 2018; Hankins et al., 2020).

Some naturally derived sulfur compounds were found to generate H_2_S in a controllable manner. Diallyl disulfide is a reactive sulfane sulfur with an unsaturated bond near the C-S. It is naturally present in garlic. Other sulfur compounds extracted from garlic extracts include cysteine alkyl disulfides, cysteine mercaptide, and diallyl trisulfide. Their antioxidant capabilities have garnered wide interests, although sometimes they displayed contradictory effects on health in clinical practice (Li et al., 2013). Recently, Cardoso et al. (2021) found that diallyl disulfide can effectively prevent emphysema induced by cigarette smoking, and this function may be exerted through modification of 4-hydroxynonenal, carbonyl reductase 1, and cytochrome P450 2E1 (CYP2E1). The diallyl disulfide can act as a histone deacetylase inhibitor and induce sustained histone hyperacetylation, thus modifying gene transcription (Nian et al., 2009). The pharmacologic potential of these reactive sulfane sulfurs in diseases accompanied by excessive oxidative stress deserves further exploration.

## Future perspective

Sulfur compounds such as H_2_S, thiol-based families, and sulfane sulfurs were fully engaged in the initiating and progressing process of COPD. They have the potential to protect individuals from developing chronic inflammatory diseases due to their antioxidant and anti-inflammation capabilities. The disturbance of antioxidant buffers, particularly low-molecular thiols, contributes to excessive oxidative stress and systemic inflammation, which may result in a high prevalence of comorbidities like cardiovascular diseases. However, the correct measurement of these sulfur compounds *in vivo* is very difficult because some of the sulfur compounds have similar chemical reactivity and they were under dynamic interchange, which arouses a major limitation during studying their bioactivities. H_2_S exhibits its function by S-sulfidation of the target protein, and the specific signaling pathway in relation to COPD deserves further exploration. Furthermore, more research studies are needed to reveal the possible relationship between sulfur compounds and comorbidities of COPD.
